# Supplementary material for: Structure of Mycobacterium tuberculosis Cya, an evolutionary ancestor of the mammalian membrane adenylyl cyclases
Source: eLife. 2022 Aug 18;11:e77032. doi: 10.7554/eLife.77032 (PMC9433096; doi:10.7554/eLife.77032)
Supplement: Supplementary file 1. [file elife-77032-supp1.docx]

**Supplementary File 1.** Cryo-EM analysis and statistics

| Instrument | FEI Titan Krios / Gatan K3 / GIF Quantum LS | |
| --- | --- | --- |
| Magnification | 130000x | |
| Voltage (kV) | 300 | |
| Electron Dose (e-/Å2) | | |
| Dataset 1 | 54 e-/Å^2^ | |
| Dataset 2 | 47 e-/Å^2^ | |
| Dataset 3 | 44 e-/Å^2^ | |
| Defocus range (μm) | -0.5 to -3.0 | |
| Pixel size (Å) | 0.66 | |
| Refinement | | |
| Number of particles | 646042 | |
| Map symmtery | **C2** | **C1** |
| Map FSC, 0.143 | 3.57 | 3.83 |
| Map sharpening b-factor (Å) | -152.693 | -155.051 |
| Model to map FSC, 0.5 | 3.9 | 4.12 |
| Map CC (mask) | 0.69 | 0.66 |
| Model composition, atoms (hydrogen atoms) | 15182 (7560) | 16928 (8413) |
| Protein residues/ligands | 990/6 | 1107/6 |
| Bond length, R.M.S.D. | 0.003 | 0.009 |
| Bond angle, R.M.S.D. | 0.642 | 1.656 |
| Validation | | |
| MolProbity score | 1.46 | 3.06 |
| Clash score | 8.5 | 23.86 |
| Rotamer outliers (%) | 0.77 | 11.24 |
| Mean B-factors protein / ligand | 46.29 / 39.85 | 83.33 / 77.95 |
| Ramachandran plot | | |
| Favoured (%) | 98.16 | 94.24 |
| Allowed (%) | 1.23 | 4.94 |
| Outliers (%) | 0.61 | 0.82 |
