# Supplementary material for: Structure of Mycobacterium tuberculosis Cya, an evolutionary ancestor of the mammalian membrane adenylyl cyclases
Source: eLife. 2022 Aug 18;11:e77032. doi: 10.7554/eLife.77032 (PMC9433096; doi:10.7554/eLife.77032)
Supplement: Supplementary file 2. [file elife-77032-supp2.docx]

**Supplementary File 2.** X-ray data analysis and statistics

|  | Cya(sol)-NB4 |
| --- | --- |
| Wavelength | 0.999879 |
| Resolution range | 47.4 - 1.973 (2.044 - 1.973) |
| Space group | P 65 2 2 |
| Unit cell | 94.793 94.793 119.655 90 90 120 |
| Total reflections | 774280 (34068) |
| Unique reflections | 21581 (1491) |
| Multiplicity | 35.9 (22.8) |
| Completeness (%) | 93.96 (66.85) |
| Mean I/sigma (I) | 25.4 (1.5) |
| Wilson B-factor | 44.58 |
| R-merge | 0.09 (1.587) |
| CC1/2 | 1.0 (0.685) |
| Reflections used in refinement | 21583 (1491) |
| Reflections used for R-free | 1077 (75) |
| R-work | 0.2056 (0.3127) |
| R-free | 0.2443 (0.3727) |
| Number of non-hydrogen atoms | 2537 |
| Macromolecules | 2325 |
| Ligands | 57 |
| Solvent | 155 |
| Protein residues | 296 |
| RMS(bonds) | 0.005 |
| RMS(angles) | 0.80 |
| Ramachandran favored (%) | 98.62 |
| Ramachandran allowed (%) | 1.03 |
| Ramachandran outliers (%) | 0.34 |
| Rotamer outliers (%) | 2.85 |
| Clashscore | 8.10 |
| Average B-factor | 58.38 |
| Macromolecules | 57.77 |
| Ligands | 63.98 |
| Solvent | 65.44 |
